# Prevalence of Doravirine Resistance Mutations in a Large-Scale HIV-1 Transmitted Drug Resistance Survey in Buenos Aires, Argentina

**DOI:** 10.3390/v17050731

**Published:** 2025-05-20

**Authors:** Diego Cecchini, Isabel Cassetti, Florencia Scarnato, Agustina Fiori, Jimena Nuevo, Clara Villaverde, Adriana Sucari, María C. Torroija, Emiliano Bissio, Gabriela Bugarin, Gustavo Lopardo

**Affiliations:** 1 Helios Salud, Buenos Aires 1141, Argentina; isabel.cassetti@gmail.com (I.C.); fscarnato@heliossalud.com.ar (F.S.); jimenanuevo@gmail.com (J.N.); 2 FUNCEI, Buenos Aires 1424, Argentina; agustinafiori@gmail.com (A.F.); mclaravillaverde@gmail.com (C.V.); glopardo@intramed.net (G.L.); 3 Laboratorio Dr. Stamboulian, Buenos Aires 1414, Argentina; asucari@stamboulian.com.ar; 4 MSD Argentina, Munro 1605, Argentina; maria.cecilia.torroija@merck.com (M.C.T.); gabriela.bugarin@merck.com (G.B.); 5 MSD Medical Affairs HIV, 28027 Madrid, Spain; emiliano.bissio@merck.com

**Keywords:** HIV, resistance, doravirine, Latin America, antiretroviral agents, prevalence, mutations

## Abstract

Background: Argentina has reported moderate to high levels of transmitted drug resistance in people living with HIV/AIDS (PLWHA), mostly to non-nucleoside reverse transcriptase inhibitors (NNRTIs). Doravirine (DOR) has a unique resistance profile and retains potent antiviral activity in the presence of the most prevalent NNRTI-associated resistant viruses. Scarce data exist regarding the frequency of DOR resistance-associated mutations (RAMs) in Latin America. We describe the prevalence of DOR RAMs in samples from adults PLWHA in Buenos Aires, Argentina, in the context of a survey of transmitted drug resistance (TDR). Material and Methods: A cross-sectional study was undertaken utilizing samples collected between 2017 and 2021 at two reference HIV clinics. Samples were analyzed for RAMs using the World Health Organization (WHO) mutation list. Mutations to DOR were assessed with the Stanford and Agence Nationale de Recherches sur le SIDA (ANRS) algorithms. Rilpivirine (RPV) RAMs were assessed using the Stanford algorithm. Susceptibility to NNRTIs was evaluated using the HIVdb Program with Stanford and ANRS criteria. Results: Samples from 1667 PLWHA were analyzed: 81.2% were male, with 52.6% identifying as men who have sex with men. According to the WHO list, the overall TDR was 12.1% (*n* = 203). The prevalence of RAMs was 10.1% (170/1667) for NNRTIs, 4% (67/1667) for nucleoside reverse-transcriptase inhibitors (NRTIs), and 1.7% (30/1667) for protease inhibitors (PIs). The most frequent NNRTI mutations were K103N (5.6%), G190A (0.89%), and K103S (0.77%). The prevalence of DOR RAMs was <2%, with the most common being Y188L (0.53%). Rilpivirine RAM prevalence was 6%. Susceptibility to DOR, RPV, efavirenz, and nevirapine as given by the Stanford algorithm was 97.4%, 92%, 91.4%, and 90.4%, respectively. The ANRS criteria yielded susceptibility rates of 98.3%, 93.3%, 92.3%, and 90.8%, respectively. Regarding NRTIs, thymidine analog mutations (including T215 revertants) were the most frequent RAMs. Among PIs, the most prevalent RAMs were M46L (0.47%) and V82A (0.35%). Conclusions: Our study shows the persistence of moderate to high levels of resistance to first-generation NNRTIs. Despite this, prevalence was low for DOR. Surveillance of TDR remains critical for recommendations of ART initiation.

## 1. Introduction

Antiretroviral therapy (ART) has revolutionized the treatment of HIV-1 infection, transforming a once-fatal disease into a manageable chronic condition [1,2]. Despite significant advancements in treatment efficacy, safety, and tolerability, challenges persist in providing access to the growing number of people living with HIV/AIDS (PLWHA) worldwide [3]. Currently, an estimated 40 million individuals are living with HIV-1 globally, with approximately 62% receiving ART [4,5]. The US Food and Drug Administration has approved over 30 antiviral compounds and 22 combination regimens for HIV-1 treatment, with non-nucleoside reverse-transcriptase inhibitors (NNRTIs) playing historically a crucial role in many treatment options [6].

As ART usage has increased, HIV-1 drug resistance has emerged as a significant global concern [7,8,9]. Resistance has developed against all antiretroviral drugs, complicating treatment selection, particularly for treatment-experienced PLWHA and in regions with high rates of transmitted drug resistance (TDR) [10,11,12]. The long-term goal of treatment is to maximize viral suppression duration using first-line therapy, delay the development of drug resistance mutations, and prevent HIV-1 transmission. However, these objectives are challenged by moderate to high primary resistance levels in certain countries [13,14].

As of 2024, about 140,000 persons were estimated to live with HIV-1 in Argentina, mainly assisted within the public health system [15]. Until recently, first-generation NNRTIs were first-line therapy for treatment initiation [15]. A comprehensive nationwide HIV-1 pretreatment drug resistance surveillance study conducted in 2019 provided crucial insights into the prevalence of drug resistance in the country [16]. This cross-sectional study, which enrolled 447 PLWHA from 19 ARV-dispensing centers, revealed a concerning increase in the frequency of resistance-associated mutations (RAMs). The study found that the overall prevalence of RAMs was 27.7% in the studied population. Notably, the prevalence of NNRTI resistance mutations was particularly high at 19.6%. For nucleoside reverse-transcriptase inhibitors (NRTIs), the prevalence was 3%, while for protease inhibitors (PIs), it was 1.5%. Among treatment-naive PLWHA, the prevalence of RAMs to first-generation NNRTIs (efavirenz and nevirapine) was 16.8%, significantly higher than previous estimates. These findings underscore the growing challenge of NNRTI resistance in Argentina and highlight the need for careful consideration when prescribing NNRTI-based regimens [16].

In response to these challenges, new treatment options and strategies are necessary. Doravirine (DOR) has emerged as a promising new NNRTI designed to address limitations of other NNRTIs, including effectiveness against common NNRTI resistance mutations (K103N, Y181C, and G190A), reduced central nervous system toxicity compared to efavirenz, and no food requirements or high baseline viral load restrictions [17,18,19,20,21]. DOR has been approved in Argentina for both ART-naive and experienced adults, either as part of a combination regimen or as a single tablet to be used with other antiretroviral drugs [22].

The high prevalence of NNRTI resistance mutations revealed by the 2019 surveillance study has significant implications for treatment guidelines in Argentina [16]. It provides evidence supporting the need for resistance testing prior to prescribing NNRTI-based regimens and supports the use of integrase strand transfer inhibitor-based ART for initial therapy. However, data on the prevalence of DOR-specific RAMs in treatment-naive populations are still lacking, making it unclear whether genotype testing is necessary before prescribing this particular drug.

To address this knowledge gap and optimize treatment strategies, we aimed to investigate the prevalence of DOR RAMs in the context of transmitted resistance to NNRTIs and other drug classes in Argentina. Our objectives are to describe, in naive PLWHA in our country, the overall prevalence of RAMs, the prevalence of DOR RAMs, the prevalence of rilpivirine RAMs, and the predicted efficacy of NNRTI drugs.

## 2. Materials and Methods

### 2.1. Study Design

The study was a retrospective cross-sectional, non-interventional study, conducted over the period 2017-09/2021. The study was conducted using only structured secondary data. Samples from ART-naive PLWHA were retrospectively analyzed for RAMS associated with resistance to DOR and other drugs at a reference laboratory (Laboratorio Dr. Stamboulian).

### 2.2. Study Population and Proceedings

This study analyzed genotype results gathered from plasma samples drawn as part of routine clinical practice from naïve adult PLWHA from two reference HIV-clinics in Buenos Aires, Argentina, ensuring systematic testing of all collected samples. Both institutions follow the guidelines from the Argentine Infectious Diseases Society (SADI) regarding care of PLWHA and ART [22].

### 2.3. Inclusion Criteria

1. Participants aged 18 years or older, at the time of genotype sampling.
2. Documented HIV-1 infection (based on a reactive Enzyme-Linked Immunosorbent Assay plus Western blot or detectable viral load).
3. Treatment-naïve, defined as no antiretrovirals (in combination or monotherapy) received before resistance test based on clinical records.

### 2.4. Exclusion Criteria

1. PLWHA without available clinical records to assure ART naïve status.
2. PLWHA with genotype assessed outside routine clinical care (e.g., clinical trial).
3. PLWHA participating in clinical trials for HIV treatment or prevention.

For characterization of study participants, the following variables were considered: age, sex, time since HIV-1 diagnosis, mode of HIV-1 acquisition, evidence of recent HIV-1 acquisition (<6 months), baseline HIV-1 RNA viral load, CD4 T-cell count (flow cytometry), and CDC HIV stage classification (assessed according to the 2014 CDC Revised Classification System for HIV-1 Infection in Adults).

All plasma samples were processed and stored for the determination of drug resistance mutations at the Laboratorio Dr. Stamboulian molecular biology division in Buenos Aires, Argentina, according to standardized procedures. Briefly, HIV-1 resistance genotyping was used for the identification of RAMs in the HIV-1 polymerase (pol) gene. The process comprises first the isolation and purification of plasma viral RNAs by MagNA Pure Compact (Roche, Basel, Switzerland), with the kit MagNA Pure Large Volume. After sample processing and RNA extraction, samples undergo an initial reverse transcription + PCR, followed by a second nested PCR step for the HIV-1 pol fragment viral region codifying for Protease and Reverse transcriptase using the Applied Biosystem (Foster City, USA), AB Veriti 96-Well Thermal Cycler. The amplified PCR products were sequenced using four primers. In the first round of PCR, the primers PRTMF1 (5′-ATGAARGATTGYACRAGRCAGGCTAAT-3′) and RTR1 (5′-ATCCCTGCATAAATCTGACTTGC-3′) were used. The second round of PCR utilized the primers PRT-F2 (5′-CTTTARCTTCCCTCARATCACTCT-3′) and RTR2 (5′-CTTCTGTATGTCATTGACAGTCC-3′). For sequencing, four primers were employed: the second-round PCR primers PRT-F2 and RTR2, along with two additional internal primers, SeqF4 (5′-CAGTACTGGATGTGGGRGAYG-3′) and SeqR4 (5′-TACTAGGTATGGTAAATGCAGT-3′). The final PCR product was 1084 base pairs in length, encompassing the protease and reverse-transcriptase regions of the HIV-1 pol gene. This fragment length provided sufficient coverage for comprehensive identification of resistance-associated mutations in both the protease and reverse-transcriptase genes, which are the primary targets for the antiretroviral drug classes analyzed in this study. The amplified PCR products were sequenced using four primers. The sequencing products were analyzed on an Applied Biosystems/Hitachi 3500 Genetic Analyzer (Waltham, MA, USA). HIV-1 subtype information was not included in this analysis, as phylogenetic characterization is not routinely performed as part of standard clinical genotyping.

The existence of a genotypic resistance test was the starting point for the development of the study. An anonymized list of genotypes in the naïve HIV-1 population was extracted and, based on laboratory ID coding/number, records were matched with the clinical records database in both institutions for extraction of aggregated clinical and epidemiological data.

The prevalence of DOR RAMs was analyzed primarily with the Stanford genotypic resistance interpretation algorithm (NNRTIs resistance notes). The following major mutations were considered: L100I, K101E, V106A/M, Y181I/V, Y188L, G190S/E, and M230L [23]. In addition, the prevalence was analyzed with the Agence Nationale de Recherches sur le SIDA (ANRS) HIV-1 list for mutations associated with resistance as follows: V106A/M, Y188L, G190E/S, M230L, L100I + K103N, K103N + Y181C, K103N + P225H, F227C and at least 4 among: A98G, L100I, K101E, V106I, E138K, Y181C/V, G190A or H221Y, L100I, K103N, K103N + Y181C, K103N + P225H, and F227C [24].

For rilpivirine (RPV), the following major mutations were considered: L100I, K101E/P, E138A/G/K/Q, Y181C/I/V, Y188L, G190A/S/E, and M230L [23]. The World Health Organization (WHO) 2009 list of mutations for surveillance of transmitted drug-resistant HIV-1 strains was used for other NNRTI, PI, and NRTI mutations [25]. The Stanford HIVdb was used to analyze RAMs according to the Stanford algorithm and ANRS criteria and to classify DOR, RPV, and first-generation NNRTIs (efavirenz and nevirapine) as susceptible, intermediate, or resistant. Sequences classified as “susceptible” and “potential low-level resistance” were grouped as “susceptible”. Etravirine was not considered in this study as not indicated for initial ART.

Viral load measurement was performed using the Cobas^®^ HIV-1 reagent, a nucleic acid test for in vitro diagnostic use on the ROCHE Cobas^®^ 4800 System. The sample type used was EDTA-preserved plasma. The assay has an analytical sensitivity of 14.2 cp/mL, a linear range of 20.0 cp/mL–1.0 × 10^0.7^ cp/mL, and 100% specificity (95% one-sided confidence interval: 99.5%). CD4-T cell counting was performed using monoclonal antibody labeling and ½ serial dilutions in whole blood. Samples were run on a BD FacsCanto II flow cytometer. Data analysis was conducted using Infinicyt 1.6 software.

Study data were collected and managed using REDCap (Research Electronic Data Capture, Vanderbilt University, Nashville, TN, USA) electronic data capture tools hosted at Fundación Helios Salud.

### 2.5. Statistical Methods

Descriptive statistics were used for the analysis of the data. Continuous variables were described using medians and interquartile ranges (IQRs). Categorical variables were described using absolute and relative frequencies. The normality of the variables was tested with Shapiro–Wilk or Kolmogorov–Smirnov tests. The absolute and relative frequency of RAMs was presented according to the drug class (NNRTI, PI, NRTI). Data were processed using Microsoft Excel (Version 16.0) and STATA 1.6 statistical software for data science.

### 2.6. Ethical Statement

The study protocol was reviewed and approved by a local ethics committee (Comité de Ética en Investigación Clínica CEIC) in Buenos Aires, Argentina (registry 6678). Due to its retrospective design, a waiver of informed consent was granted as the research involved no direct patient contact.

## 3. Results

### 3.1. Study Population Characteristics

A total of 1677 genotypes from PLWHA classified as ART-naïve were recorded. The demographic, clinical, and immuno-virological profiles are detailed in Table 1. The distribution of patients was as follows: 68.5% from Helios Salud and 31.5% from FUNCEI. Briefly, the majority of participants were young males, having sex with men being the predominant route of HIV acquisition. Most cases were asymptomatic, and approximately 10% showed evidence of HIV acquisition within the prior six months. For the mutation analysis, 1667 genotypes were included, as 10 did not have available results.

### 3.2. Transmitted Drug Resistance Mutations According to WHO List

**Figure 1:**
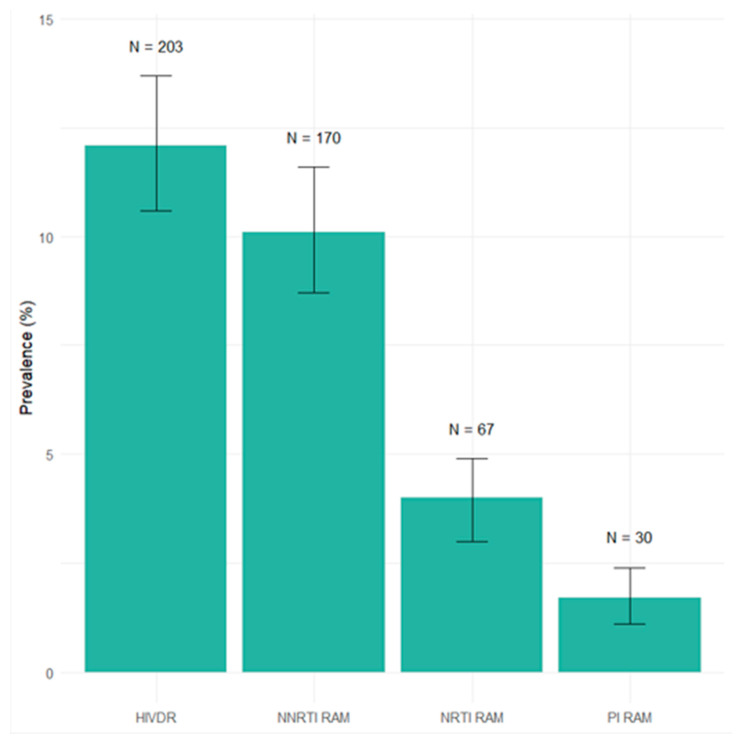
Prevalence of transmitted drug resistance (WHO list) in PLWHA who underwent genotype testing in Buenos Aires, Argentina (period 2017–2021). HIVDR: HIV drug resistance mutations; NNRTI: non-nucleoside reverse transcriptase inhibitor; NRTI: nucleoside reverse-transcriptase inhibitor; PI: protease inhibitor; PLWHA: people living with HIV/AIDS.

**Figure 2:**
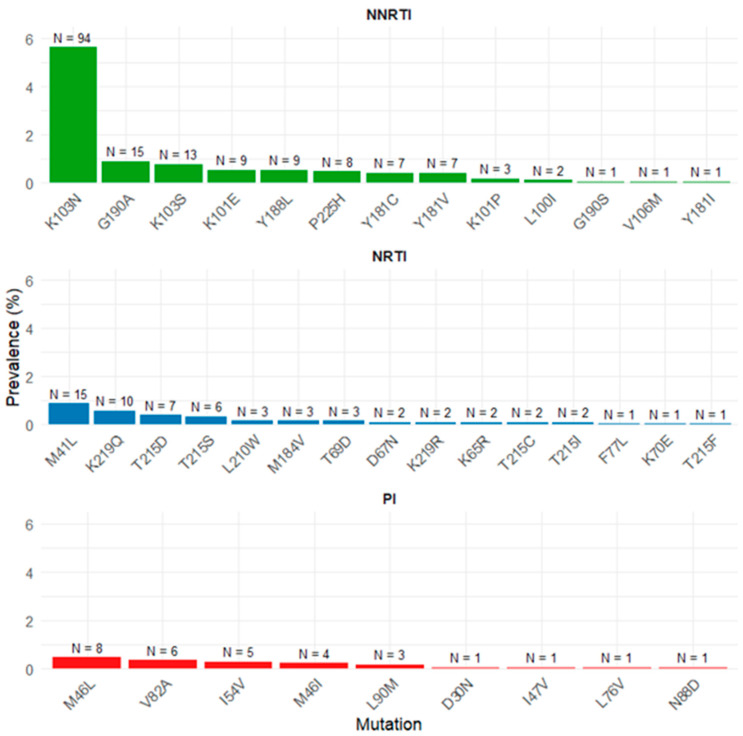
Prevalence of individual drug resistance-associated mutations (WHO list) in 1667 PLWHA who underwent genotype testing in Buenos Aires, Argentina (period 2017–2021). Values are numbers (percentages). NNRTI: non-nucleoside reverse-transcriptase inhibitor; NRTI: nucleoside reverse-transcriptase inhibitor; PI: protease inhibitor; PLWHA: people living with HIV/AIDS.

Considering the WHO list, at least one mutation associated with transmitted drug- resistant HIV-1 strains was detected in samples from 203 individuals, resulting in a TDR prevalence of 12.1%. The prevalence of NNRTI-associated mutations was 10.1% (170/1667), the prevalence of NRTI RAMs was 4% (67/1667), and the prevalence of PI RAMs was 1.7% (30/1667), as shown in Figure 1. The most frequent NNRTI mutations were K103N, G190A, and K103S. Regarding NRTIs, thymidine analog mutations (including T215 revertants) were the most frequent RAMs, observed in 3.3% of cases followed by M184V (0.17%). Among PIs, the most prevalent RAMs were M46L (0.47%) and V82A (0.35%). The list of RAMs is described in Figure 2.

### 3.3. Transmitted Drug Resistance to Doravirine

According to the Stanford HIVDR algorithm, 30 (1.79%) samples harbored at least one DOR-RAM, being the most prevalent Y188L (0.53%) and K101E (0.53%). A detail of DOR-RAMs is shown in Table 2.

Considering the ANRS algorithm, 23 individual mutations or combinations of 2 or 4 RAMs were detected: 22 were individual mutations or a combination of 2 mutations and one case of the combination of four RAM criteria, providing an overall prevalence of 1.37% (Table 3).

### 3.4. Transmitted Drug Resistance to Rilpivirine

Among 101 participants (6%), a total of 109 major mutations associated with RPV resistance were described (mostly at expense of the E138A polymorphism). A detail is shown in Table 4. Also, two cases with A98G and V179L (0.11% each) and three of H221Y (0.17%), which may impact on RPV susceptibility, were described.

### 3.5. Predicted Efficacy of NNRTI Drugs

Analysis using the Stanford algorithm showed susceptibility rates to DOR, RPV, efavirenz, and nevirapine of 97.9%, 92%, 91.4%, and 90.4%, respectively. The ANRS criteria yielded similar but slightly higher susceptibility rates: 98.3% for DOR, 93.3% for RPV, 92.3% for efavirenz, and 90.8% for nevirapine. Regarding high-level resistance to DOR, the Stanford algorithm classified 0.6% of sequences in this category, while the ANRS algorithm identified 1.07% as resistant.

## 4. Discussion

Transmitted drug resistance has been a major issue in Latin American countries, mostly due to resistance to first-generation NNRTIs [16,26,27,28,29]. This could be attributable to several factors, including their frequent prescription as first-line therapy until recent years, low genetic barrier, and persistence of resistant variants within viral quasispecies due to lack of impact on viral fitness [30]. The region reflects dynamic epidemiological trends, with regional studies indicating a moderate overall prevalence of 7.7% (2000–2015) and a significant rise from 6.0% to 8.2% between 2000 and 2005 and 2006 and 2015 [30]. This increase is driven by growing NNRTI resistance (e.g., K103N, G190A), while NRTI resistance declined, likely due to shifts in antiretroviral therapy regimens. Sub-regional disparities exist: Brazil, Mesoamerica, and the Southern Cone show NNRTI-dominated TDR, whereas the Caribbean exhibits rapid increases across all drug classes, including protease inhibitors [30]. Acute/recent HIV-1 infections in Brazil reveal heightened NNRTI resistance (16.3%), underscoring risks to efavirenz-based first-line therapies [28]. Monitoring TDR is critical given uneven ART rollout, evolving national guidelines, and variable viral suppression rates, which shape resistance transmission. Sustained surveillance enables early detection of emerging mutations, informs regimen updates (integrase inhibitor adoption), and mitigates compromised treatment efficacy [26].

Evidence of cross-resistance within the NNRTI drug class has been widely described in the literature [31], so drug resistance mutations to first-generation NNRTIs may impact the susceptibility of later-generation NNRTIs, limiting future treatment options. DOR is a novel NNRTI approved both for initiating ART and in switch strategies. Regarding the initiation of treatment, DOR appears as recommended in the European AIDS Clinical Society (EACS) guidelines, whereas in others it is regarded as recommended in certain clinical situations (U.S. Department of Health and Human Services, DHHS) [32,33]. However, the potential preexistence of RAMs is a major concern as NNRTI resistance has been widely described in Latin American countries as well as in epidemiological surveys from Europe and the USA [30,34,35,36]. In this context, updated information regarding the prevalence of TDR considering not only first-generation NNRTIs but also DOR is needed.

In Argentina, the SADI recommends second-generation integrase inhibitors (INSTIs) as preferred anchor drugs for initial ART without requiring a baseline genotype test. Regarding DOR, SADI considers it an alternative option for which a baseline genotype is recommended but not mandatory [22]. This approach aligns with the drug’s resistance profile and the current understanding of TDR in Argentina. To date, there are no data about pretreatment resistance to DOR within the country. Furthermore, updated data regarding the prevalence of TDR to NNRTIs and other drug classes have been lacking in recent years in Latin America.

Considering the number of samples analyzed, our study is, to date, the largest survey of TDR in Argentina and has important epidemiological and clinical implications. First, it shows evidence of the persistence of moderate levels of TDR according to the WHO mutation list, mostly in the NNRTI class. This is in concordance with prior studies. Bissio et al. reported in a multicentric country-representative survey for the period 2014–2015 a 13% prevalence of TDR, 10% for the NNRTI drug class, mostly due to the K103N mutation [27]. A previous report from one of the institutions involved in this study showed a 12.1% point prevalence of TDR among 91 PLWHA during 2011–2013. Seven mutations corresponded to NNRTIs, four to nucleoside analogs, and two to protease inhibitors. The most frequent RAMs were K103N and M41L [37]. Other reports in pregnant women and perinatally infected newborns also showed moderate to high levels of TDR [29,38,39,40]. The more recent study by Laufer et al. provides further evidence of its evolution in Argentina: the study found an overall prevalence of RAMs of 27.7%, with 19.6% prevalence for NNRTIs. This represents a significant increase from the previous survey [27], particularly in NNRTI resistance. For treatment-naïve individuals, the prevalence of NNRTI RAMs was 16.8% [16]. These findings underscore the growing concern about NNRTI resistance and support the shift towards INSTI-based regimens as first-line treatment in the country. In light of these findings, our study shows stable moderate levels of TDR despite first-line therapeutic options moving towards other drug classes with a higher genetic barrier (INSTIs) in recent years.

Second, considering potential cross-resistance between NNRTI drugs, our study also shows stable moderate levels of TDR to RPV, about 6%. This is in accordance with the 8% previously reported in a countrywide survey [41] but higher than the 3% described previously by one of the participating institutions [37]. In this context, a genotypic test before initiation of rilpivirine-based therapy should be considered.

Third, our research is unique for the region in terms of providing the first exploratory data regarding resistance to DOR in the naïve population. The existence of mutations or combinations conferring resistance to DOR should not be attributable to the rollout of this drug in Argentina as it is not recommended for first-line ART in local guidelines and not available in the public health system [22]. Therefore, it may be considered a consequence of cross-resistance with first-generation NNRTIs that have been widely used. Our study, using two different algorithms, shows low levels of TDR to this drug, below the 5% threshold for considering the cost-effectiveness of performing a genotype before starting therapy [42,43]. No data within the region have been presented to date for a fair comparison; however, international reports tend to support our results. Two studies in Europe (France and Poland) showed low levels of DOR RAMs: Soulie et al. described a 1.4% prevalence among 9764 sequences, and Scheibe et al. reported RAMs in 32 patients (1.6%) out of 1960 sequences analyzed using bulk sequencing, of whom 13 (0.74%) were naïve and 19 (9.50%) were treatment-experienced [44,45]. A study in South Africa with samples from 277 patients initiating ART showed a prevalence <5%, while a study in Botswana reported a moderate prevalence [46]. Overall, published studies tend to show low levels of RAMs for this drug, but further research is needed.

Considering the predicted efficacy according to both the Stanford and ANRS algorithms described here, approximately 98% of samples demonstrated susceptibility to DOR, while only about 1% could be classified as resistant. These results consistently show a higher susceptibility to DOR compared to other NNRTIs. This finding is reassuring and supports DOR as a viable option for initiating ART without genotype testing. The high susceptibility rate suggests that DOR maintains its effectiveness in settings with moderate to high levels of TDR to first-generation NNRTIs.

Despite the strength provided by the high number of samples included, our study has several important limitations that should be addressed. First, the cross-sectional design limits our ability to assess long temporal trends in resistance patterns and does not allow for follow-up evaluation of clinical outcomes. Second, it was conducted by two private institutions from the city of Buenos Aires, providing services for the health-insured population, which introduces potential selection bias. This may limit the extrapolation of our results to PLWHA assisted in the public health system, who represent the majority of cases in Argentina and may have different resistance profiles. Third, our study is geographically restricted to Buenos Aires and neighboring suburbs: despite the HIV epidemic being mostly concentrated in the Buenos Aires metropolitan area, our results may not reflect the state of resistance prevalence in other districts or provinces [15]. Fourth, our study was performed based on real-life genotype reports, which do not routinely provide HIV subtype information. The laboratory workflow prioritizes the detection and reporting of clinically relevant resistance-associated mutations rather than comprehensive phylogenetic analysis. Therefore, subtype-specific analysis was beyond the scope of this particular study. Although the usual subtypes described in Argentina are B and B/F [29], we are not able to address the prevalence or influence of HIV-1 genetic diversity on the resistance mutation patterns described here. This is a major limitation, as genetic variants can vary in their biological properties, evolutionary rates, disease progression, and resistance mutation profiles, potentially affecting the interpretation of our findings.

Despite most ART recommendations, including SADI guidelines, having shifted to non-efavirenz therapies as first-line regimens, surveillance of TDR remains a key issue to improve ART prescription policies within the naïve population [22,32]. Our study demonstrates the persistence of moderate to high levels of TDR, mostly in the NNRTI class, without compromising the predicted efficacy of DOR for starting ART. To the best of our knowledge, this is the largest DOR resistance study in naive PLWHA carried out so far and may impact local and regional guidelines supporting the initiation of DOR-based therapy without a baseline genotype. This may potentially impact its position within national guidelines for the treatment-naive population and contribute to expanding therapeutic options in the country. This information significantly contributes to the pursuit of effective, accessible treatment for all affected PLWHA, especially in the context of rising NNRTI resistance rates.

## Figures and Tables

**Table 1:** Baseline characteristics of 1677 ART-naïve PLWHA who underwent genotype testing in Buenos Aires, Argentina (period 2017–2021). Values are numbers (percentages) unless otherwise stated (participants with available data).

Variable	N, (%)
Age (median, IQR)	34 (28–43)
Gender	
Male	1356 (81.2)
Female	318 (19)
Transgender	3 (0.17)
Route of infection (*n* = 1442)	
Sexual	
Heterosexual	681 (47.23)
MSM	759 (52.6)
Other	2 (0.14)
Viral load (copies/mL), median (IQR)	50,550 (15,300–183,500)
CD4 T-cell count (cell/uL), median (IQR)	363 (212–532)
Recent infection *	166 (9.97)
Clinical category (*n* = 1670)	
Asymptomatic	1263 (75.6)
Class B symptoms	139 (8.3)
Class C symptoms	173 (10.3)
Acute retroviral syndrome	95 (5.63)

MSM: men who have sex with men; PLWHA: people living with HIV/AIDS; * acquired <6 months.

**Table 2:** Prevalence of individual drug resistance-associated mutations to doravirine (Stanford genotypic resistance interpretation algorithm) in 1667 PLWHA who underwent genotype testing in Buenos Aires, Argentina (period 2017–2021). Values are numbers (percentages).

	**N (%)**
K101E	9 (0.53)
Y188L	9 (0.53)
Y181V	7 (0.41)
L100I	2 (1.17)
V106M	1 (0.05)
G190S	1 (0.05)
Y181I	1 (0.05)

PLWHA: people living with HIV/AIDS.

**Table 3:** Prevalence of drug resistance-associated mutations to doravirine (ANRS algorithm) in 1667 PLWHA who underwent genotype testing in Buenos Aires, Argentina (period 2017–2021). Values are numbers (percentages).

	N (%)
Y188L	9 (0.53)
K103N + P225H	6 (0.35)
K103N + Y181C	3 (0.17)
L100I + K103N	2 (0.11)
G190S	1 (0.05)
V106M	1 (0.05)
At least 4 of A98G, L100I, K101E, V106I, E138K,	
Y181C, Y181V, G190A or H221Y, L100I, K103N,	
K103N + Y181C, K103N + P225H, F227C	1 (0.05)

PLWHA: people living with HIV/AIDS.

**Table 4:** Prevalence of individual major drug resistance-associated mutations to rilpivirine (Stanford genotypic resistance interpretation algorithm) in 1667 PLWHA who underwent genotype testing in Buenos Aires, Argentina (period 2017–2021). Values are numbers (percentages).

Mutations Associated with Possible Resistance	N (%)
E138A	64 (3.83)
E138G	9 (0.53)
Y188L	8 (0.47)
Y181C	7 (0.41)
E138K	7 (0.41)
K101E	5 (0.29)
Y181V	5 (0.29)
K101P	3 (0.17)
E138Q	1 (0.05)

PLWHA: people living with HIV/AIDS.

## Data Availability

The datasets generated and/or analyzed during the current study are available from the corresponding author(s) on reasonable request.
